# Cervical Intraepithelial Lesions in Women with Persistent Inflammatory Smear on Pap Smear: A Descriptive Cross-sectional Study

**DOI:** 10.31729/jnma.7011

**Published:** 2021-09-30

**Authors:** Junu Shrestha, Dilasma Gharti Magar, Chandani Pandey

**Affiliations:** 1 Department of Obstetrics and Gynaecology, Manipal Teaching Hospital; 2 Department of Pathology, Manipal Teaching Hospital, Pokhara, Nepal

**Keywords:** *cervix*, *colposcopy*, *inflammation*, *pap smear*, *squamous intraepithelial lesion*

## Abstract

**Introduction:**

Persistent inflammatory smear is a benign finding on pap test but is associated with premalignant lesion of the cervix. Further evaluation is therefore necessary. This study was done to determine the prevalence of cervical intraepithelial lesions in women with persistent inflammatory smear.

**Methods:**

This is descriptive observational study conducted in Department of Obstetrics and Gynaecology of a tertiary care centre of Nepal from 15th May 2020 to 14th May 2021 after obtaining ethical clearance from Institutional Review Board (Reference no MEMG/IRC/338/GA). Women with two consecutive pap smear reports showing inflammatory findings were enrolled. Colposcopy was performed and Modified Reid's colposcopic index was used to grade the lesions. Colposcopic guided biopsy was taken and tissue sent for histopathology for abnormal colposcopic lesions. Data analysis was done using Statistical Package for Social Sciences version 21. Point estimate at 95% Confidence Interval was calculated along with frequency and proportion for binary data.

**Results:**

Among 115 women, 57 (49.5%) at 95% Confidence Interval (40.37-58.63) had cervical intraepithelial lesions. Among them 48 (41.7%) had low grade intraepithelial lesions and 9 (7.8%) had high grade lesions on colposcopy.

**Conclusions:**

The prevalence of cervical intraepithelial lesions in women with persistent inflammatory smear on pap was higher in our study compared to other studies.

## INTRODUCTION

Inflammatory smear is one of the common findings noted in pap smear.^1-3^ Cervical inflammation is mostly due to infections.^3-4^ However it may occur following chronic irritation from foreign body, trauma and chemical irritants.^5^ Persistent inflammatory smear is when two consecutive smears reports show inflammatory changes without atypia. Though a benign finding in pap smear, persistent inflammatory smears have been found to be associated with preneoplastic changes.^6-9^ Deferring further evaluation with colposcopy and guided biopsy whenever necessary may delay timely diagnosis of precancerous lesions in many.

Cervical cancer is the second most common cancer in Nepal.^10^ Hence, timely diagnosis and treatment of precancerous cervical lesions would help in decreasing the number of new cases of cervical cancer. Therefore, evaluation of persistent inflammatory smear with colposcopy followed by biopsy is an important secondary screening tool.

Therefore, this study was conducted with the aim to determine the prevalence of cervical intraepithelial lesions in women with persistent inflammatory smear on pap smear.

## METHODS

This was a descriptive cross-sectional study conducted in the outpatient department of Obstetrics and Gynaecology Department of Manipal Teaching Hospital, Pokhara, Nepal for a period of one year from 15th May 2020 to 14th May 2021. Ethical approval was taken from the Institutional Review Committee of Manipal Teaching hospital prior to conducting the study (Reference no MEMG/IRC/338/GA). Convenient sampling method was used. The study included all the women with two consecutive pap smear showing inflammatory findings without features of atypia. These women had been treated with antibiotics after the first smear showed inflammatory findings. Repeat smear was performed after six to twelve weeks of treatment and if repeat smear showed inflammatory findings, they were included in the study. Pregnant women, women on follow up after treatment for cervical cancer or premalignant lesion and women with diabetes mellitus, HIV were excluded. Women refusing to participate in the study were also excluded.

Sample size calculation was done using the formula,

n = Z^2^ × p × q / e^2^

  = Z^2^ × p × (1-p) / e^2^

  = (1.96)^2^ × 0.16 × (1 - 0.84) / 0.07^2^

  = 105

where,

Z = 1.96 for 95% confidence interval
p = 16%, prevalence of cervical intraepithelial neoplasia in women with persistent inflammatory smear from previous study 16%^6^
q = 1-p
e = margin of error 7%

Minimum sample size of 105 was calculated. However, a total of 115 participants were included in the study. The participants were informed about the nature of the study and informed consent was obtained prior to enrolment. Detailed history was taken in relation to age at marriage, menstrual history, parity and any specific gynaecological complaints. General and systemic examination was done and they were subjected for colposcopy.

Digital video colposcope (HD Inttelio) BORZE-USA, was used for this procedure. The colposcope was adjusted till the focal length was obtained for optical resolution. The cervix was visualized after moistening it with normal saline. Gross lesions, vascular details and opacity of the epithelium were looked for at this examination in low to medium magnification (4 to 10 times). Cervix was then viewed with a green filter to study vascular configuration of tissue. High magnification (10 to 20X) was used for visualization of the vascular abnormalities. Presence of any vascular changes and their nature were viewed in detail. Subsequently, the cervix was treated with freshly prepared solution of 5% acetic acid (prepared as 1ml acetic acid and 19 ml of normal saline) applied with cotton swab for one minute. Any change in the cervical epithelium was noted. The intensity of the acetowhite areas, margins, borders and contour of the lesion were noted. When necessary 5% acetic acid was reapplied and cervix visualized. Finally, the cervix was treated with commercially available Lugol's iodine and inspected. Change in colour of the cervical tissue was noted after this. The Modified Reid's colposcopic index was used for the interpretation of colposcopic findings. The Reid's colposcopic index considers four colposcopic signs - margin of the lesion, color of acetowhite lesion, blood vessels, and staining with Lugol's iodine.^11^ Cervix which had no acetowhite areas, vascular changes or staining Mahogany brown with Lugol's iodine were considered normal. If the RCI score was 0 to 2, it was reported as Low Grade Intraepithelial Lesion (LSIL), 3 to 4 as overlap between LSIL and High Grade Intraepithelial Lesion (HSIL) and score 5 to 8 as HSIL. Modified RCI was used because other studies have shown good correlation of modified RCI with the histopathological findings.^12,13^

The decision to take a biopsy depended on the discretion of the person performing colposcopy. If deemed necessary, biopsies were still taken from normal and benign looking cervix during colposcopy. Ifabnormalities were detected in colposcopy, targeted biopsies were taken with Baby Tischler punch biopsy forceps. All the tissues biopsied were fixed in 10% formalin and sent to the pathology department for examination. The findings of histopathology were followed up and recorded.

All the information was recorded in the preformed proforma. Data entry and analysis was done using the latest Statistical Package for Social Sciences (SPSS) software version 21. Data were presented using frequencies and percentages. Point estimate at 95% Confidence Interval was calculated.

## RESULTS

Colposcopic reporting was done according International Federation for Cervical Pathology and Colposcopy (IFCPC).^14^ On colposcopy, 57 (49.5%) (40.37-58.63 at 95% Confidence Interval) had abnormal colposcopic findings, mostly low grade squamous intraepithelial lesion (LSIL) and benign findings were noted in 30 (26.1%) women. Colposcopy was normal in 28 (24.3%) women. In six of them, biopsy was not taken (Table 1).

**Table 1:** Colposcopic findings of patients with persistent inflammatory smear on Pap (n = 115).

Colposcopic Findings		n (%)
Normal colposcopic findings		28 (24.3)
Abnormal colposcopic findings	LSIL	48 (41.7)
	HSIL	9 (7.8)
	Invasive carcinoma	0 (0)
Miscellaneous findings	Immature squamous metaplasia	26 (22.6)
	Cervicitis	4 (3.5)

On comparison of the findings of colposcopy with that of histopathology, reports of colposcopy and histopathology matched in 42 (75%) with normal or benign findings, 32 (68.1%) cases with LSIL and 3 (60%) cases with HSIL. In 10 (21.3%) participants with normal colposcopy, histopathology showed LSIL. Likewise, 13 (23.2%) patients who had LSIL in colposcopy, histopathology report came as normal or benign disorders (Table 2).

**Table 2:** Histopathological diagnosis among cervical intra-epithelial lesions (n = 57).

Abnormal colposcopie findings	Histopathological diagnosis				
	Normal/Benign	LSIL	HSIL	N/A^*^	Total
LSIL	13 (23.2)	32 (68.1)	2 (40)	1 (14.3)	48 (41.7)
HSIL	1 (1.8)	5 (10.6)	3 (60)	0(0)	9 (7.8)

* Not available/Not done

There were 115 participants in the study. The mean age of the patients was 42 years and mean years at marriage was 22 years. Majority 80 (69.3%) of the patients had a regular menstrual cycle. Most of them were multiparous. Per vaginal discharge was the common complaint with which patients had presented. On a gross naked eye examination, half of the patients had a healthy looking cervix and the other half had a clinically unhealthy-looking cervix (Table 3).

**Table 3:** Clinical characteristics ofthe participantswith persistent inflammatory smear on pap (n = 115).

Mean age (years)	42.3 (±9.0)
Mean age at marriage (years)	22.1 (± 9.2)
Clinical Characteristics	n (%)
Menstrual cycles	
Regular	80 (69.6)
Irregular	16 (13.9)
Postmenopausal	19 (16.5)
Intermenstrual bleeding	0 (0)
**Parity**	
Nulliparous	2 (1.7)
Primiparous	8 (7.0)
Multiparous	98 (85.2)
Grand multiparous	7 (6.1)
**Symptoms at Presentation**	
Routine	14 (12.1)
Per vaginal discharge	91 (79.1)
Postmenopausal bleeding	1 (0.9)
Intermenstrual bleeding	1 (0.9)
**Appearance of cervix on examination**	
Healthy	58 (50.4)
Unhealthy	57 (49.6)

## DISCUSSION

Inflammatory smear as a finding in pap smear is quite common but there is no consensus to the further treatment. Treatment with antibiotics is considered in some places but in some, it is left on its own considering it as a benign condition. It has been a known fact that persistent inflammation leads to increased cellular changes and metaplasia, further increasing the chance of malignant transformation.^15^ Hence, purpose of this study was to find the prevalence of precancerous lesions of cervix in women with persistent inflammatory smear.

The mean age of the women in this study was 42 years similar to that reported by Hmid B et al. and Dasari P et al.^8,9^ Other studies reported lesser age around 30 years as mean age of patients.^6,7,16^ Another study reported almost 50% of women belonging to 20 to 30 years age group.^17^ The difference in the mean age could be due to different study settings and different screening practice in countries where studies have been conducted. Higher mean age in our study could be because women were getting screened at a later age than in other countries. The mean age of marriage was 22 years in our study similar to that of another study.^6^

Persistent inflammatory smear was more common in multiparous women. Same was the finding of other studies.^7,8,16,17^ This is expected outcome as multiparity is a known risk factor for premalignant and malignant lesions of cervix.

Vaginal discharge was the common symptom of the women with persistent inflammatory smear; same as reported by the others.^8,16^ However, 12% of women with repeated inflammation on pap test got tested as a routine test.

In this study, almost 49.5% women had abnormal colposcopic findings; 41.7% had low grade lesion and 7.8% had high grade lesions. Colposcopy was normal in 24% of women. In 26.1% women colposcopic finding was that of immature squamous metaplasia and cervicitis. Similarly, in some other studies, almost 50 % of the study participants had abnormal colposcopic findings.^6,17^ Colposcopic findings were normal in 24% of women with persistent inflammatory smear in our study compared to 6 to 10% in studies conducted by Dasari P et al. and Hmid R et al.^8,9^

This disparity in the normal and abnormal colposcopy could be due to different study population included in the study and could also be because of different criteria used to label colposcopy as normal or abnormal. Lack of uniformity in using standard nomenclature and classification has made comparisons difficult and unreliable.

On colposcopy, nearly 50% of women had cervical intraepithelial neoplasia. Other studies also reported abnormal colposcopy ranging from 50 to 64% women with persistent inflammatory smear.^6,8,16,17^ Almost 42% women were diagnosed with low grade lesion and 7.8% with high grade lesion on colposcopy. Higher number of women had high grade lesion (20%) was reported by Nahar K et al.^17^ However, Shanmugham D et al. reported that 44% of women had low grade lesion and 18% had high grade lesion on colposcopy. ^16^ Hence, high grade lesion on colposcopy was lower in our study compared to other studies.

In six patients, biopsy was not taken as colposcopy was normal and one patient had inadequate tissue and hence was not included in the analysis. Of 108 women who had colposcopic guided biopsy, 45% women had premalignant lesions - LSIL in 40.7 % women and HSIL in 4.3% women. There were no cases of microinvasive lesion or invasive carcinoma. Chronic cervicitis was found in 43.5% women almost the same as that reported by Bhutia K et al. and Shanmugham D et al. ^6,16^ The proportion of cases with premalignant lesion diagnosed on histopathology in this study was comparatively higher than in other studies.^6,9,16,17^ Low grade lesions were more frequent than the high grade lesions. The proportion of women with high grade lesions was 4.3% and there were no cases of invasive carcinoma in our study. Dasari P et al. report similar proportion of high grade lesions but there were also few cases of carcinoma in situ and invasive carcinoma.^8^ Other studies reported high number of cases of high grade lesions in women with persistent inflammatory smear.^16,17^

On analyzing the colposcopic diagnosis with histopathological diagnosis, correlation between two was present in 75% of normal/benign findings, 68% cases of low grade lesions and 60% cases of high grade lesions in the present study. Colposcopy though normal in 21% cases, histopathology revealed that there was a low grade lesion. Likewise, 23% cases diagnosed as LSIL and one case diagnosed as HSIL on colposcopy was found to have benign findings on histopathology. Hence, taking colposcopy guided biopsy in all cases may minimize the false negative and false positive findings in patients undergoing further investigation for persistent inflammatory smear.

This study was conducted at a single centre and did not include a diverse group of participants. Convenient sampling was used in this study. Hence the findings of the study cannot be generalized to a larger population. This was an observational study, analysis of the correlation between the findings of colposcopy and histopathology could not be done to see the sensitivity and specificity of colposcopy. Hence, a larger study involving multiple centres with analytical study design would yield more useful results.

## CONCLUSIONS

The prevalence of cervical intraepithelial lesion in women with persistent inflammatory smear on the pap test was higher in our study compared to other studies. Hence, evaluation of women with persistent inflammatory smear with colposcopy and biopsy is recommended. This will help in early diagnosis and treatment of cervical precancerous lesions.
